# Utility of ^18^F-Fluorodeoxyglucose Positron Emission Tomography in Inflammatory Rheumatism, Particularly Polymyalgia Rheumatica: A Retrospective Study of 222 PET/CT

**DOI:** 10.3389/fmed.2020.00394

**Published:** 2020-08-13

**Authors:** Julie Amat, Marion Chanchou, Louis Olagne, Lucie Descamps, Anthime Flaus, Clément Bouvet, Bertrand Barres, Clemence Valla, Ioana Molnar, Arnaud Cougoul, Sylvain Mathieu, Olivier Aumaitre, Martin Soubrier, Antony Kelly, Charles Merlin, Florent Cachin

**Affiliations:** ^1^Jean Perrin Oncology Institute, Department of Nuclear Medicine, Clermont-Ferrand, France; ^2^Gabriel Montpied University Hospital, Department of Internal Medicine University of Clermont-Ferrand, Clermont-Ferrand, France; ^3^Gabriel Montpied University Hospital, Department of Rheumatology University of Clermont-Ferrand, Clermont-Ferrand, France; ^4^North University Hospital, Department of Nuclear Medicine, University of Saint-Etienne, Saint-Etienne, France; ^5^Jean Perrin Oncology Institute, Department of Biostatistics, Clermont-Ferrand, France

**Keywords:** PMR, ^18^F-FDG PET/CT, inflammatory rheumatism, uptake scores, SUVmax, bursa

## Abstract

**Purpose:** The objective of this study was to evaluate periarticular FDG uptake scores from ^18^F-FDG-PET/CT to identify polymyalgia rheumatica (PMR) within a population presenting rheumatic diseases.

**Methods:** A French retrospective study from 2011 to 2015 was conducted. Patients who underwent ^18^F-FDG-PET/CT for diagnosis or follow-up of a rheumatism or an unexplained biological inflammatory syndrome were included. Clinical data and final diagnosis were reviewed. Seventeen periarticular sites were sorted by a visual reading enabling us to calculate two scores: mean FDG visual uptake score, number of sites with significant uptake same as that or higher than liver uptake intensity and by a semi-quantitative analysis using mean maximum standardized uptake value (SUVmax). Optimal cutoffs of visual score and SUVmax to diagnose PMR were determined using receiver operating characteristics curves.

**Results:** Among 222 ^18^F-FDG PET/CT selected for 215 patients, 161 ^18^F-FDG PET/CT were performed in patients who presented inflammatory rheumatism as a final diagnosis (of whom 57 PMR). The presence of at least three sites with significant uptake identified PMR with a sensitivity of 86% and a specificity of 85.5% (AUC 0.872, 95% CI [0.81–0.93]). The mean FDG visual score cutoff to diagnose a PMR was 0.765 with a sensitivity of 82.5% and a specificity of 75.8% (AUC 0.854; 95% CI [0.80–0.91]). The mean SUVmax cutoff to diagnose PMR was 2.168 with a sensitivity of 77.2% and a specificity of 77.6% (AUC 0.842; 95% CI [0.79–0.89]).

**Conclusions:** This study suggests that ^18^F-FDG PET/CT had good performances to identify PMR within a population presenting rheumatic diseases.

## Key points

-**Question:** The objective of our study was to evaluate visual and semi-quantitative periarticular scores derived from ^18^F-FDG PET/CT for the diagnosis of PMR among rheumatic diseases.
-**Pertinent Findings:** This retrospective study showed that the presence of at least three sites with visual significant uptake and a mean SUVmax at the 17 sites equal to or greater than 2.168 had high sensitivities and specificities for the diagnosis of PMR.
-**Implications For Patient Care:** An accuracy ^18^F-FDG PET/CT periarticular analysis guides the clinician when the clinical presentation is atypical, especially in cases of rheumatism in the elderly and, therefore, has an impact on early therapeutic management.

## Introduction

Chronic inflammatory rheumatisms are common conditions among the general population. Rheumatoid arthritis (RA) is the most frequent rheumatism in France, with a prevalence of 0.35% (1). In people older than age 50, the prevalence of polymyalgia rheumatica (PMR) and giant cell arteritis (GCA) is 700/100,000 and 204/100,000, respectively (2).

The ACR/EULAR's 2010 criteria for RA (3) and its 2012 criteria for PMR (4) enable orientation of the diagnosis of these diseases; however, their sensitivities and specificities remain limited (57.9% and 88.8% for RA, 66% and 81% for PMR, respectively).

Moreover, the need to eliminate differential and associated diagnoses, such as neoplasias and vasculitis (5, 6) especially in elderly people, encourages additional examinations. Fluorodeoxyglucose positron emission tomography coupled with computerized tomography (^18^F-FDG PET/CT) in these cases seems useful. Macrophage activation and fibroblast proliferation enhanced by proinflammatory cytokines result in an increased fluorodeoxyglucose (^18^F-FDG) uptake in articular, periarticular, and vascular wall areas (7). Inflammation targets the synovial membrane in patients suffering from RA. In cases of PMR, it affects principally the serous bursa. Several studies demonstrated the usefulness of ^18^F-FDG PET/CT in inflammatory rheumatism diseases (5, 6, 8–10), especially PMR and in vasculitis. ^18^F-FDG PET/CT enables a full-body map of vascular, articular, and periarticular uptake within a single examination (9, 10).

Several scores have been developed for the diagnosis of vasculitis or inflammatory rheumatism and to evaluate their activity (11–13) with relatively promising results.

The objective of our study was to evaluate composite periarticular scores derived from ^18^F-FDG PET/CT for the diagnosis of PMR among rheumatic diseases.

## Materials and Methods

### Patients

In this retrospective study, 478 patients were selected. Their ^18^F-FDG PET/CT were performed between April 2011 and December 2015 and prescribed by the Rheumatology and Internal Medicine Departments of our institution (Clermont-Ferrand, France).

^18^F-FDG PET/CT inclusion criteria were follow-up of previously known rheumatic diseases such as PMR, RA, GCA, and spondyloarthritis (SA); diagnosis of suspected rheumatic diseases; and diagnosis of an unexplained biological inflammatory syndrome.

The following data were collected when available: indication of the ^18^F-FDG PET/CT (initial test for inflammatory rheumatism or for an unexplained biological inflammatory syndrome, test for treatment resistance, screening for vasculitis, or a neoplasia), rheumatism's activity parameters such as C-reactive protein (CRP), erythrocyte sedimentation rate (ESR), DAS28-VS or DAS28-CRP, treatment with corticosteroids or other immunosuppressants (including duration and dose), and final diagnosis retained by a rheumatologist or an internal medicine specialist according to the patient's clinical and paraclinical data. ^18^F-FDG PET/CT exams were not included in the paraclinical tests used for the final rheumatic diagnosis. In the majority, ^18^F-FDG PET/CT was realized to rule out paraneoplastic rheumatism.

In case of an unclassified rheumatism, diagnosis was applied according to the 2010 ACR/EULAR's criteria for RA (3), its 2012 criteria for PMR (4), and the 2009 ASAS's criteria for SA (14). If the rheumatism did not meet these criteria, a final diagnosis was agreed upon collegially by the three principal investigators. Some patients remained with a diagnosis of unclassified rheumatism.

Patients were sorted into four groups:

- The group named “inflammatory rheumatisms” gathered patients with PMR, RA, SA, GCA, microcrystalline rheumatism, synovitis-acne-pustulosis-hyperostosis-osteitis (SAPHO), unclassified rheumatism, remitting seronegative symmetrical synovitis with pitting edema (RS3PE), paraneoplastic rheumatism, and psoriatic rheumatism.
- The group named “rheumatic diseases without inflammatory rheumatism” referred to patients who ultimately presented discopathy, vertebral collapse, prosthetic loosening, narrowing of the lumbar vertebral canal, tendinitis of the gluteus medius muscle, fracture of the pelvis, shoulder–hand syndrome, fibromyalgia, or osteoarthritis.
- The group named “infectious or inflammatory diseases” gathered a majority of patients addressed for an unexplained biological inflammatory syndrome and who ultimately displayed infectious or inflammatory diseases, some of whom did not have musculoskeletal manifestations.
- The group named “absence of inflammatory rheumatism” included patients in the groups named “rheumatic diseases without inflammatory rheumatism” and “infectious or inflammatory diseases.”

Eighty percent of GCA cases were proven histologically with a positive temporal biopsy. For the others, the diagnosis was based on clinical and paraclinical data (imaging).

The patients provided their written informed consent to participate in this study.

The study has been approved by CECIC Rhône Alpes Auvergne, Grenoble, IRB 5921 on 12 November 2019 (IRB number: 5921).

### ^18^F-FDG PET/CT Imaging

After 4 h of fasting, a minimal activity of 3 MBq/kg of ^18^F-FDG was injected into a peripheral vein. Acquisition was achieved 1 h after injection on a PET/CT scanner (Discovery ST or Discovery 710 Optima 660). In most cases, acquisition extended from the skull to the upper third of the femurs, with the upper extremities situated either along the body or above the head. Only 15% of the ^18^F-FDG PET/CT involved the entire body. ^18^F-FDG PET/CT acquisitions were not contrast-enhanced.

Similar to Sondag et al.'s (9) method, 17 periarticular sites were analyzed using a visual analysis to evaluate the intensity and the number of hotspots. A semi-quantitative analysis was also realized for the 17 hotspots. These involved both shoulders, both acromioclavicular and both sternoclavicular joints, the most intense interspinous bursa, both hips, both greater trochanters, both ischial tuberosities, both iliopectineal bursa, and both symphysis pubis enthesis. Each uptake was sorted by visual analysis using a four-point scale from 0 to 3 in comparison with liver uptake (0: no uptake, 1: uptake lower than the liver, 2: moderate uptake, same as that of the liver, 3: uptake higher than the liver).

Two visual composite scores were therefore analyzed: the mean FDG uptake score at the 17 sites of an exam: *F*_17_ and the number of sites with significant uptake [score ≥ 2, cutoff proposed by Goerres et al. (15): Nb].

Moreover, the maximum standardized uptake value (SUVmax) was measured at the 17 hotspots for 222 ^18^F-FDG PET/CT by a board-certified nuclear medicine physician, blinded to the clinical and paraclinical test results, using Advantage Windows Server 3.2 (General Electric Healthcare Systems, 2016). For determination of the SUVmax, a region of interest (ROI) was manually placed over each of the 17 periarticular sites. Activity concentration within the ROI was determined and expressed as SUV, where SUV is the ratio of the activity in the tissue to the decay corrected activity injected into the patient and normalized for patient body weight. SUVmax was used as the reference measurement and was determined by considering the uptake given by the maximum pixel value within a ROI in each of the 17 hotpsots.

For PMR, RA, and all pathologies taken together, ^18^F-FDG PET/CT was sorted into two groups (<3 sites with significant uptake, ≥3 sites with significant uptake) in order to compare the rheumatism's activity parameters.

### Statistical Analysis

Parameters were calculated and then compared within the different groups (PMR, RA, SA, GCA, all inflammatory rheumatisms taken together, “absence of inflammatory rheumatism,” and “rheumatic diseases without inflammatory rheumatism”):

- Mean FDG uptake score (*F*_17_) and standard deviation,
- Number of sites with significant uptake (Nb) and standard deviation,

The means of the scores were compared using the Kruskal-Wallis test.

Some other parameters were calculated and compared between the PMR ± GCA group and other patients as follows using the Wilcoxon-Mann-Whitney test:

- Mean SUVmax for each of the 17 hotspots,
- Mean SUVmax for the 17 hotspots.

The sensitivity and specificity for the diagnosis of PMR were calculated using ROC curve.

Rheumatism's activity parameters (CRP, duration and dose of corticotherapy, DAS28-VS or DAS28-CRP) as well as age were calculated and compared based on the presence or absence of three sites with significant uptake for the groups PMR, RA and all pathologies taken together. The latter were compared using Student's test or the Kruskal-Wallis test. A bilateral *p*-value lower than the cutoff of 0.05 was considered statistically significant.

## Results

### Patients

**Figure 1:**
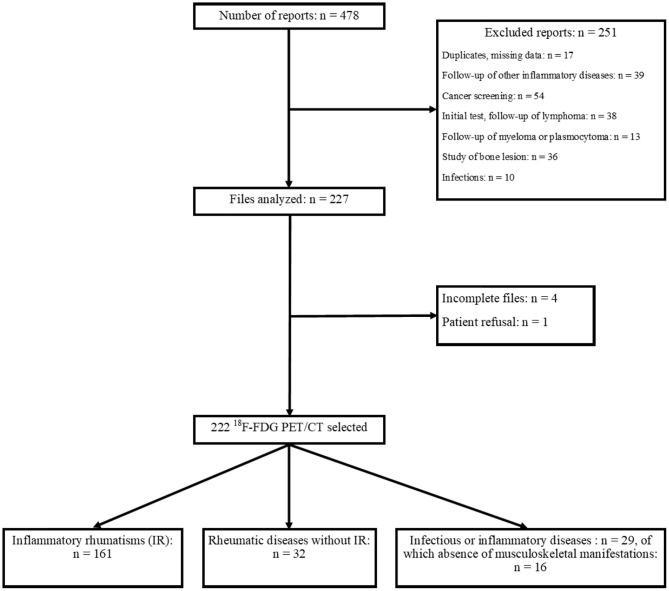
Flowchart.

Overall, 222 ^18^F-FDG PET/CT were selected for 215 patients as part of the testing for rheumatic diseases, vasculitis, neoplasias, or the exploration of an unexplained biological inflammatory syndrome. A flowchart is displayed in Figure 1.

Distribution of ^18^F-FDG PET/CT according to the final diagnosis and characteristics of the population is given in Tables 1, 2. Table 2 summarizes the characteristics of the patients of the PMR group: median age 74.8 years (IQR 15.2), 31 women, 26 men, CRP 32 mg/L (IQR 66), 13 mg/day (IQR 5.8) corticosteroids. CRP values and corticosteroid dose were, respectively, available for 53 and 30 patients of the PMR group. In our whole population, these values were used, respectively, for 90 and 57 patients.

**Table 1:** Distribution of ^18^F-FDG PET/CT according to the final diagnosis.

**Final Diagnosis**	**Number of ^**18**^F-FDG PET/CT**	**%**
PMR	57	25.7
Of which, PMR + GCA	*10*	4.5
GCA (without PMR)	10	4.5
RA	49	22.1
SA	18	8.1
Psoriatic rheumatism	5	2.2
SAPHO	3	1.4
RS3PE	4	1.8
Paraneoplastic rheumatism	4	1.8
Microcrystalline rheumatism	5	2.2
Unclassified rheumatism	6	2.7
Rheumatic diseases without inflammatory rheumatism	32	14.4
Infectious or inflammatory diseases	29	13.1
Of which, patients without musculoskeletal manifestations	*16*	7.2
**Total**	222	100

**Table 2:** Characteristics of the population.

**Characteristics**	**All patients**	**PMR ± GCA**
Gender, *n* (%)		
Men	89/215 (41.4)	26/57 (45.6)
Women	126/215 (58.6)	31/57 (54.4)
Age, median (IQR), years	70.4 (20.5)	74.8 (15.2)
CRP, median (IQR), mg/L	16 (49.6)	32 (66)
Steroids dose, median (IQR), mg/day	10 (9)	13 (5.8)
**Total**	215	57

### ^18^F-FDG PET/CT Visual Analysis

A visual score was calculated for 17 periarticular sites based on liver uptake comparison.

Summarized in Table 3, the mean FDG uptake score at the 17 sites (*F*_17_) was significantly higher in the group of PMRs compared with the group “absence of inflammatory rheumatism,” respectively, 1.32 ± 0.61 and 0.44 ± 0.31 (*p* < 10^−7^). Likewise, the number of sites with significant uptake (Nb) was also higher, respectively, 6.9 ± 4.88 and 0.62 ± 1.2 (*p* < 10^−7^) (Table 3).

**Table 3:** Results of the different visual composite scores (m ± s^a^) according to the final diagnosis.

**Parameters**	**All IRs^**b**^ taken together** **(*n* = 161)**	**PMR^**c**^** **± GCA** **(*n* = 57)**	**RA^**d**^** **(*n* = 49)**	**SA^**e**^** **(*n* = 18)**	**GCA^**f**^** **without PMR** **(*n* = 10)**	**Absence** **of IR** **(*n* = 61)**	**Rheumatic diseases without IR** **(*n* = 32)**
*F*${}_{17}^{\text{g}}$	0.88 ± 0.63	1.32 ± 0.61	0.65 ± 0.41	0.69 ± 0.67	0.32 ± 0.31	0.44 ± 0.31	0.45 ± 0.29
Nb^h^	3.52 ± 4.47	6.90 ± 4.88	1.53 ± 2.18	2.56 ± 4.34	0.3 ± 0.67	0.62 ± 1.20	0.62 ± 1.13

a *m ± s, Mean and standard deviation*.

b *IR, Inflammatory rheumatism*.

c *PMR, Polymyalgia rheumatic*.

d *RA, Rheumatoid arthritis*.

e *SA, Spondyloarthritis*.

f *GCA, Giant cell arteritis*.

g *F_17_ is the mean FDG uptake score studied in the 17 sites*.

h *Nb is the number of sites with significant uptake (≥ liver uptake)*.

For the PMR diagnosis, the predictive cutoff values of the mean FDG uptake score (*F*_17_) and the number of sites with significant uptake were determined, respectively, at 0.765 (sensitivity of 82.5%, specificity of 75.8%, AUC 0.854; 95% CI [0.80–0.91]) and ≥3 (sensitivity of 86% and specificity of 85.5%, AUC 0.872, 95% CI [0.81–0.93]).

Impairment of at least three sites (Nb ≥ 3) and a mean FDG uptake score >0.765 (*F*_17_ > 0.765) appeared to be the most specific criteria (respectively, 85.5 and 75.8%) for identifying PMR.

**Figure 2:**
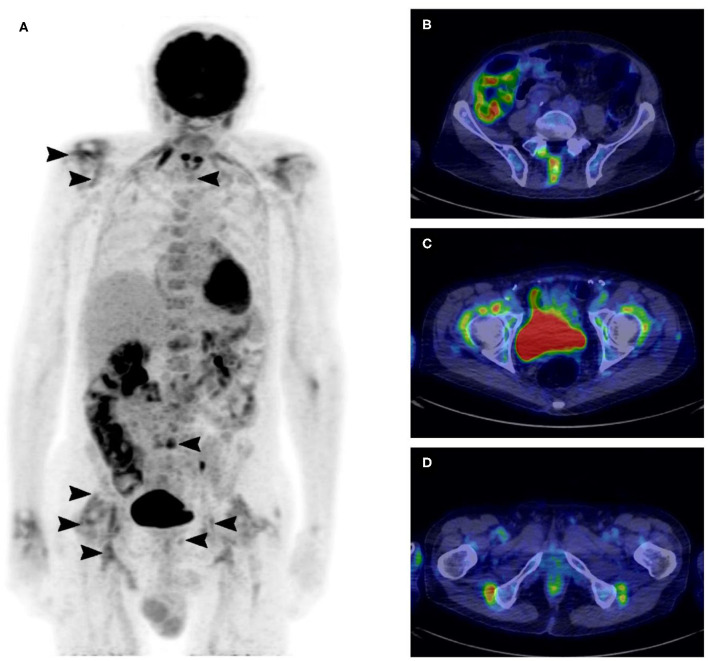
^18^F-FDG PET/CT maximum-intensity projection showing uptake at the 17 sites of the skeleton in a patient suffering from polymyalgia rheumatica **(A)**, axial fused ^18^F-FDG PET/CT with typical uptake of polymyalgia rheumatica at the lumbar interspinous bursa **(B)**, at the iliopectineal bursa **(C)**, and at the ischial bursa **(D)**.

For example, this maximum-intensity projection and the axial fused ^18^F-FDG PET/CT show a patient suffering from PMR with uptake at the 17 sites in Figure 2.

### Relationship Between Visual FDG Uptake and Rheumatism Activity

Rheumatism activity parameters were calculated and compared in some groups according to the number of sites with visual uptake equal or superior to the liver background, which was the most sensitive and the more specific score. Results are shown in Table 4.

**Table 4:** Rheumatism activity parameters (m ± s^a^) based on the number of sites with significant uptake on ^18^F-FDG PET/CT for PMR, RA, and all pathologies taken together.

**Parameters**	**All pathologies taken together**			**RA**^**b**^			**PMR**^**c**^ **±** **GCA**^**d**^		
	**PET** **Nb^***e***^ ≥ 3** **(*n* = 73)**	**PET** **Nb <3** **(*n* = 149)**	***P*^**f**^**	**PET** **Nb ≥ 3** **(*n* = 10)**	**PET** **Nb <3** **(*n* = 39)**	***p***	**PET** **Nb ≥ 3** **(*n* = 48)**	**PET** **Nb <3** **(*n* = 9)**	***p***
CRP^g^ (mg/L)	58.6 ± 60.9	29.9 ± 46	<10^−5^	74.0 ± 49.1	32.9 ± 53.2	0.0065	53.4 ± 61.7	43.1 ± 55.9	0.54
CT^h^ duration (months)	NA^i^			32.4 ± 78.8	35.4 ± 68.6	0.94	21.7 ± 53.1	6.7 ± 11.7	0.95
CT dose (mg/day)				2.9 ± 4.2	3.7 ± 5.7	0.9	5.9 ± 6.6	11.3 ± 10.0	0.18
Age (in years)	70.8 ± 12.2	64.4 ± 21.2	0.034	65.4 ± 14	64.8 ± 13.2	0.80	72.9 ± 10.9	76.7 ± 6.7	0.36
DAS28^j^	NA			6.0 ± 1.3	4.1 ± 1.3	0.0045	NA		

a *m ± s, Mean and standard deviation*.

b *RA, Rheumatoid arthritis*.

c * PMR, Polymyalgia rheumatica*.

d *GCA, Giant cell arteritis*.

e * Nb is the number of sites with significant uptake (≥ liver uptake)*.

f *p, Significance value p*.

g *CRP, C-reactive protein*.

h *CT, Corticosteroids*.

i * NA, Not applicable*.

j *DAS 28, Disease Activity Score 28*.

CRP values of RA and “all pathologies taken together” groups were significantly higher in patients who had at least three sites with significant uptake on their exams (respectively, *p* = 0.0065 and *p* < 10^−5^). Likewise, DAS 28 in the RA group was significantly higher (6.0 ± 1.3 vs. 4.1 ± 1.3 with *p* = 0.0045).

Patients belonging to the group “all pathologies taken together” were older when there were at least three sites with significant uptake on ^18^F-FDG PET/CT (*p* = 0.034).

Finally, we did not find any significant link between the dose and duration of corticosteroid use in patients with PMR or RA based on the number of sites with significant uptake.

### ^18^F-FDG PET/CT Semi-Quantitative Analysis

SUVmax was measured on each of the 17 periarticular sites (Table 5). The mean SUVmax at the 17 sites was significantly higher in the PMR ± GCA group compared with the others, respectively, 2.68 (±0.63) and 1.81 (±0.69) (*p* < 10^−6^).

**Table 5:** Results of the mean SUVmax (m ± s^a^) on ^18^F-FDG PET/CT according to the final diagnosis.

**Parameters**	**All PET/CT except PMR^b^± GCA^c^** **(*N* = 165)**	**PMR^b^± GCA^c^** **(*N* = 57)**
	**Mean SUVmax**	**Mean SUVmax**
Right sternoclavicular	1.93 ± 0.83	2.61 ± 0.87
Left sternoclavicular	1.89 ± 0.82	2.56 ± 0.75
Right acromioclavicular	1.84 ± 0.82	2.56 ± 0.84
Left acromioclavicular	1.89 ± 0.94	2.51 ± 0.81
Right glenohumeral	2.15 ± 1.17	3.13 ± 1.09
Left glenohumeral	2.11 ± 1.12	2.96 ± 0.99
Interspinous bursa	1.95 ± 0.99	3.13 ± 1.44
Right iliopectineal bursa	1.57 ± 0.75	2.48 ± 0.8
Left iliopectineal bursa	1.68 ± 1	2.61 ± 1.12
Right hip	1.78 ± 0.87	2.61 ± 0.95
Left hip	1.93 ± 1.16	2.75 ± 1.11
Right symphysis pubis enthesis	1.53 ± 0.63	2.4 ± 0.67
Left symphysis pubis enthesis	1.54 ± 0.69	2.5 ± 0.73
Right greater trochanter	1.72 ± 0.82	2.52 ± 0.84
Left greater trochanter	1.68 ± 0.69	2.63 ± 1.08
Right ischial tuberosity	1.74 ± 0.94	2.78 ± 0.97
Left ischial tuberosity	1.76 ± 0.94	2.86 ± 1.07
17 hotspots	1.81 ± 0.69	2.68 ± 0.63

a *m ± s, Mean and standard deviation*.

b *PMR, Polymyalgia rheumatic*.

c *GCA, Giant cell arteritis*.

**Figure 3:**
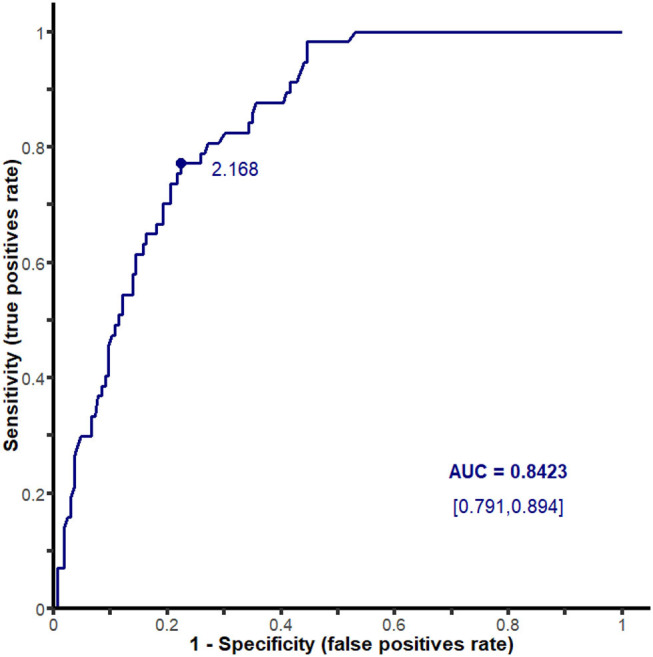
ROC curve analyzing ^18^F-FDG PET/CT performance for the diagnosis of polymyalgia rheumatica according to the mean SUVmax at the 17 sites.

The predictive cutoff of the mean SUVmax at the 17 sites for PMR was calculated at 2.168 (sensitivity of 77.2%, specificity of 77.6%, AUC 0.842; 95% CI [0.79–0.89]) (Figure 3).

Moreover, these results were also significantly higher in each of the 17 sites for the PMR ± Horton group.

The best predictive mean SUVmax cutoff to diagnose a PMR was determined at 2.168 (sensitivity of 77.2%, specificity of 77.6%, AUC 0.842; 95% CI [0.79–0.89]).

### ^18^F-FDG PET/CT Visual and Semi-Quantitative Analysis

The sensitivities and specificities of four composite scores (*F*_17_ > 0.53, *F*_17_ > 0.765, Nb ≥ 3, SUVmax ≥ 2.168) enabling diagnosis of the studied diseases are given in Table 6.

**Table 6:** Sensitivities (Se) and specificities (Sp) of the different composite scores (*F*_17_ > 0.53, *F*_17_ > 0.765, Nb ≥ 3, SUVmax > 2.168) based on the final diagnosis (all inflammatory rheumatisms taken together, PMR, RA, SA, absence of inflammatory rheumatism, and rheumatic diseases without inflammatory rheumatism).

**Parameters**	**%**	**All** **IRs^a^** **(*n* = 161)**	**PMR^b^± GCA^c^** **(*n* = 57)**	**RA^d^** **(*n* = 49)**	**SA^e^** **(*n* = 18)**	**Absence** **of IR** **(*n* = 61)**	**Rheumatic diseases without IR** **(*n* = 32)**
*F*${}_{17}^{\text{f}}$ > 0.53	Se	61.5	91.2	51	33.3	32.8	34.4
	Sp	67.2	59.4	45.7	44.6	38.5	43.2
*F*_17_ > 0.765	Se	48.4	82.5	32.7	33.3	14.8	15.6
	Sp	85.2	75.8	59	60.3	51.6	56.8
Nb^g^≥ 3	Se	42.9	86	20.4	27.8	6.6	6.2
	Sp	93.4	85.5	63.6	66.7	57.1	62.6
SUVmax^h^≥ 2.168	Se	42.2	77.2	20.4	27.8	21.3	21.9
	Sp	78.7	77.6	59	62.7	57.8	61.1

a *IR, Inflammatory rheumatism*.

b *PMR, Polymyalgia rheumatic*.

c *GCA, Giant cell arteritis*.

d *RA, Rheumatoid arthritis*.

e *SA, Spondyloarthritis*.

f *F_17_ is the mean FDG uptake score studied in the 17 sites*.

g *Nb is the number of sites with significant uptake (≥ liver uptake)*.

h *SUVmax is the mean SUVmax for the 17 hotspots*.

## Discussion

### Key Findings of the Study and Comparison to the Literature

To date, our study, with 222 ^18^F-FDG PET/CT analyzed, has been one of the largest in terms of evaluating ^18^F-FDG PET/CT in cases of inflammatory rheumatism. Visual and semi-quantitative analyses were realized on 17 periarticular sites. Also, our work consolidates various rheumatic diseases beyond cases of PMR, as was also done by Yamashita et al. (11), who included cases of PMR, RA, and SA. Yamashita et al. (11) demonstrated the usefulness of scores when categorizing cases of PMR from other rheumatic diseases (particularly RA and SA), by analyzing uptake in ischial tuberosities, in greater trochanters, and in interspinous bursa. Compared with the SA group, the ratio of FDG uptake was significantly higher in patients with PMR and lower in patients with RA in ischial tuberosities (63.2, 93.8, and 12.5%, respectively; *p* < 0.001), greater trochanters (47.4, 81.3, and 12.5%; *p* < 0.001), and interspinous bursa (52.63, 75.0, and 12.50%; *p* = 0.001). Likewise, in our study, the number of sites with significant uptake (Nb) was also higher in the PMR group compared to RA or SA, respectively, 6.9 ± 4.88, 1.53 ± 2.18, and 2.56 ± 4.34 (*p* < 10^−7^) (Table 3).

Wakura et al. (12) used uptake scores in nine articular and periarticular sites (scapulohumeral and coxofemoral joints, greater trochanters, ischial tuberosities, interspinous bursa at the cervical, thoracic and lumbar levels, entheses of the pectineal muscle and the right femoral muscle) within two groups, PMR (15 patients) and EORA (seven patients). The uptake scores allowed differentiation between the cases of PMR and EORA, with the PMR group showing statistically significant higher scores. They also compared the SUVmax for abnormal FDG accumulation sites between the PMR and EORA patients and observed no significant differences between the two groups. Takahashi et al. (13) compared five articular sites uptake between PMR and EORA patients. They found a sensitivity of 92.6% and a specificity of 90% in favor of PMR when three out of the following five items were present: uptake greater than that of the liver in the shoulders, interspinous bursa, iliopectineal bursa, and ischial tuberosities associated with the absence of uptake in the wrists. In the PMR group, the results were statistically higher in the ischial tuberosities and interspinous bursa; however, the uptake was lower in the wrists. Concerning our study, the number of sites with significant uptake was higher in PMR patients than in the RA group, 6.90 ± 4.88 and 1.53 ± 2.18, respectively (*p* < 10^−7^).

In order to diagnose PMR, our study found sensitivity and specificity values of 86 and 85.5%, respectively, when the ^18^F-FDG PET/CT presented at least three sites with significant uptake (Nb ≥ 3), which is higher than the results found by Sondag et al. (9) with a sensitivity of 74% and a specificity of 79% for a score Nb ≥ 3.

We found a significant link between the visual uptake intensity, an elevated CRP, and older age in the “all pathologies taken together” group when the ^18^F-FDG PET/CT found at least three sites with significant uptake (*p* ≤ 0.01). Sondag et al. (9), Moosig et al. (16), and Okamura et al. (17) also found that CRP rates were correlated to the uptake intensity in patients with PMR or vasculitis. We found a correlation between the intensity and number of periarticular uptake (at least three sites with significant uptake) and a higher DAS 28 score in patients with RA (6.0 ± 1.3 vs. 4.1 ± 1.3 with *p* at 0.0045), which was also described by Okamura et al. [15]. On the other hand, we did not highlight any significant link among the presence of at least three sites with significant uptake and the dose and duration of corticosteroids in the PMR and RA groups. This may be explained by the fact that, in our study, the rheumatism's activity parameters had not been noted on the day of the ^18^F-FDG PET/CT. However, Blockmans et al. found a decreased uptake in the joints of the axial skeleton after 3 months of corticosteroids in 35 patients suffering from PMR (18) and in the vascular walls after 3 months of corticosteroids in 35 patients with GCA (19).

Blockmans et al. (18) did not recommend performing a ^18^F-FDG PET/CT when following up cases of PMR because the decreased uptake was correlated to biological results. A more recent study evaluated the use of ^18^F-FDG PET/CT for the assessment of tocilizumab as first-line treatment in PMR patients (20). FDG uptake and bioclinical parameters (physical examination, CRP, and ESR) after treatment were significantly decreased. However, the correlation between SUVmax and the other bioclinical parameters was low. This result may be explained by the low level of SUVmax variation compared to that of the other parameters. SUVmax was significantly decreased in all regions except in the shoulders, sternoclavicular joints, and cervical interspinous bursa. This persistent FDG uptake should be explained by joint remodeling during the few weeks after tocilizumab treatment. In our study, a large majority of patients (158) were free from any corticotherapy or immunosuppressive treatments at the time of ^18^F-FDG PET/CT acquisitions guaranteeing the absence of any induced treatment modification of ^18^F-FDG accumulation in joint sites. For the others, presence or absence of corticotherapy or immunosuppressive treatments was not clearly recorded in data files.

Our study show that the visual score is more sensitive and more specific than the semi-quantitative score (sensitivity of 86% and specificity of 85.5% when at least three sites had a significant uptake and sensitivity of 77.2% and specificity of 77.6% when the mean SUVmax at the 17 sites was equal to or greater than 2.168). Moreover, the visual score is easier to use in daily practice.

Approximately 20% of patients with apparently isolated PMR showed LVV on ^18^F-FDG PET/CT (21). As PMR and GCA are frequently overlap, typical FDG joint uptake patterns and vascular uptake should be reported using a standardized 0-to-3 grading system (no uptake ≼ mediastinum, low < liver, intermediate = liver, high > liver), (21–23) with grade 2 considered as possibly positive for active LVV and grade 3 positive for active LVV (23). Moreover, Slart et al. highlighted that ^18^F-FDG PET/CT exhibited high diagnostic performance for the detection of LVV and PMR and was able to evaluate the response to treatment (17, 23, 24).

### Limitations and Strengths of the Study

This study has a few limitations. One concerns missing data relating to the study's retrospective design. In this monocenter study, inclusion criteria were heterogeneous. Indeed, patients with various rheumatic diseases such as PMR, RA, SA, psoriatic rheumatism, and microcrystalline rheumatism were included and ^18^F-FDG PET/CT were achieved either for initial diagnosis (to search for vasculitis or neoplasia) or during the follow-up (after treatment resistance). Moreover, acquisition methods were heterogeneous, performed on two different PET/CT systems leading to quantitative differences. In addition, ^18^F-FDG PET/CT were analyzed by the same observer, which creates doubts concerning its reproducibility, which was not assessed in our study. However, the use of a four-point scale, according to four intensity levels from 0 to 3, in comparison with liver uptake, and SUVmax values enable this variability to be reduced. This visual method was already used in the Deauville score for therapeutic evaluation of lymphomas (25).

The large number of patients included especially PMR ones and the visual and semi-quantitative assessments are part of the strengths of the study.

### Integration Into the Current Understanding and Future Direction of the Research

The ^18^F-FDG PET/CT allows us to confirm and map periarticular inflammation. Therefore, it is an exam to be prioritized in clinically contentious cases, especially rheumatism in elderly patients (26).

Infections, neoplasias, and the different rheumatic diseases can reproduce the same musculoskeletal symptoms. The importance of early diagnosis enables initiation of the proper treatment and reduction of anatomical and functional sequels.

Therefore, it is important to refine the reading of ^18^F-FDG PET/CT by precisely indicating the number and intensity of the periarticular uptake. This allows the clinician to be guided toward a diagnosis when the clinical presentation is atypical, especially in cases of rheumatism in the elderly and, therefore, to have an impact on therapeutic management.

A prospective study should be realized to confirm these results.

## Conclusion

The visual and semi-quantitative scores turned out to be effective in differentiating PMR from another rheumatism with a sensitivity of 86% and a specificity of 85.5% when at least three sites had a significant uptake and a sensitivity of 77.2% and a specificity of 77.6% when the mean SUVmax at the 17 sites was equal to or greater than 2.168.

## Data Availability Statement

All datasets generated for this study are included in the article/supplementary material.

## Ethics Statement

The studies involving human participants were reviewed and approved by CECIC Rhône Alpes Auvergne, Grenoble, IRB 5921. The patients/participants provided their written informed consent to participate in this study.

## Author Contributions

MC and AF especially contributed to acquiring data. LO and LD contributed to conception and design. CB, BB, and CV contributed to revising the manuscript and approval of the final content of the manuscript. IM and AC contributed to interpreting data. SM, OA, MS, CM, AK, and FC contributed to enhancing the intellectual content. All authors contributed to the article and approved the submitted version.

## Conflict of Interest

The authors declare that the research was conducted in the absence of any commercial or financial relationships that could be construed as a potential conflict of interest.

## Abbreviations

ACR/EULAR: American College of Rheumatology/European League Against Rheumatism
ASAS: Assessment of Spondyloarthritis International Society
AUC: Area under curve
BASDAI: Bath Ankylosing Spondylitis Disease Activity Index
CRP: C-reactive protein
CT: Corticotherapy
DAS28-VS or DAS28-CRP: Disease Activity Score
EORA: Elderly-onset rheumatoid arthritis
ESR: Erythrocyte sedimentation rate
^18^F-FDG PET/CT: Fluorodeoxyglucose positron emission tomography coupled with computerized tomography
GCA: Giant cell arteritis
IR: Inflammatory rheumatism
LVV: Large vessel vasculitis
MBq/kg: Megabecquerel per kilogram body weight
PMR: Polymyalgia rheumatica
RA: Rheumatoid arthritis
ROC: Receiver operating characteristic
ROI: Region of interest
RS3PE: Remitting symmetrical seronegative synovitis with pitting edema
SA: Spondyloarthritis
SAPHO: Synovitis-acne-pustulosis-hyperostosis-osteitis
SUVmax: Maximum standardized uptake value
TAB: Temporal artery biopsy.

## References

[1] Fautrel B Cukierman G Joubert J-M Laurendeau C Gourmelen J Fagnani F Characteristics and management of rheumatoid arthritis in France: analysis of a representative French national claims database resulting in an estimated prevalence of 0.35%. Jt Bone Spine. (2016) 83:461–2. 10.1016/j.jbspin.2015.05.010 26678000

[2] Crowson CS Matteson EL. Contemporary prevalence estimates for giant cell arteritis and polymyalgia rheumatica, 2015. Semin Arthritis Rheum. (2017) 47:253–6. 10.1016/j.semarthrit.2017.04.001 28551169 PMC5623160

[3] Cornec D Varache S Morvan J Devauchelle-Pensec V Berthelot J-M Le Henaff-Bourhis C. Comparison of ACR 1987 and ACR/EULAR 2010 criteria for predicting a 10-year diagnosis of rheumatoid arthritis. Jt Bone Spine. (2012) 79:581–5. 10.1016/j.jbspin.2012.01.015 22405855

[4] Dasgupta B Cimmino MA Maradit-Kremers H Schmidt WA Schirmer M Salvarani C. 2012 provisional classification criteria for polymyalgia rheumatica: a European League Against Rheumatism/American College of Rheumatology collaborative initiative. Ann Rheum Dis. (2012) 71:484–92. 10.1136/annrheumdis-2011-200329 22388996 PMC3298664

[5] Dalkiliç E Tufan AN Hafizoglu E Hafizoglu M Tufan F Oksuz F. The process from symptom onset to rheumatology clinic in polymyalgia rheumatica. Rheumatol Int. (2014) 34:1589–92. 10.1007/s00296-014-3034-y 24816791

[6] Lavado-Pérez C Martínez-Rodríguez I Martínez-Amador N Banzo I Quirce R Jiménez-Bonilla J. (18)F-FDG PET/CT for the detection of large vessel vasculitis in patients with polymyalgia rheumatica. Rev Esp Med Nucl Imagen Mol. (2015) 34:275–81. 10.1016/j.remnie.2015.07.005 26159505

[7] Matsui T Nakata N Nagai S Nakatani A Takahashi M Momose T. Inflammatory cytokines and hypoxia contribute to 18F-FDG uptake by cells involved in pannus formation in rheumatoid arthritis. J Nucl Med. (2009) 50:920–6. 10.2967/jnumed.108.060103 19443596

[8] Yamashita H Kubota K Mimori A. Clinical value of whole-body PET/CT in patients with active rheumatic diseases. Arthritis Res Ther. (2014) 16:423. 10.1186/s13075-014-0423-2 25606590 PMC4289312

[9] Sondag M Guillot X Verhoeven F Blagosklonov O Prati C Boulahdour H. Utility of 18F-fluoro-dexoxyglucose positron emission tomography for the diagnosis of polymyalgia rheumatica: a controlled study. Rheumatology. (2016) 55:1452–7. 10.1093/rheumatology/kew202 27107429

[10] Elzinga EH van der Laken CJ Comans EFI Lammertsma AA Dijkmans BAC Voskuyl AE. 2-Deoxy-2-[F-18]fluoro-D-glucose joint uptake on positron emission tomography images: rheumatoid arthritis versus osteoarthritis. Mol Imaging Biol. (2007) 9:357–60. 10.1007/s11307-007-0113-4 17902022 PMC2040173

[11] Yamashita H Kubota K Takahashi Y Minamimoto R Morooka M Kaneko H. Similarities and differences in fluorodeoxyglucose positron emission tomography/computed tomography findings in spondyloarthropathy, polymyalgia rheumatica and rheumatoid arthritis. Joint Bone Spine. (2013) 80:171–7. 10.1016/j.jbspin.2012.04.006 22749663

[12] Wakura D Kotani T Takeuchi T Komori T Yoshida S Makino S. Differentiation between Polymyalgia Rheumatica (PMR) and elderly-onset rheumatoid arthritis using 18F-fluorodeoxyglucose positron emission tomography/computed tomography: is enthesitis a new pathological lesion in PMR? PLoS ONE. (2016) 11:e0158509. 10.1371/journal.pone.0158509 27384410 PMC4934779

[13] Takahashi H Yamashita H Kubota K Miyata Y Okasaki M Morooka M. Differences in fluorodeoxyglucose positron emission tomography/computed tomography findings between elderly onset rheumatoid arthritis and polymyalgia rheumatica. Mod Rheumatol. (2015) 25:546–51. 10.3109/14397595.2014.978936 25401232

[14] Rudwaleit M Van Der Heijde D Landewé R Listing J Akkoc N Brandt J. The development of assessment of SpondyloArthritis international Society classification criteria for axial spondyloarthritis (part II): validation and final selection. Ann Rheum Dis. (2009) 68:777–83. 10.1136/ard.2009.108233 19297344

[15] Goerres GW Forster A Uebelhart D Seifert B Treyer V Michel B. F-18 FDG whole-body PET for the assessment of disease activity in patients with rheumatoid arthritis. Clin Nucl Med. (2006) 31:386–90. 10.1097/01.rlu.0000222678.95218.42 16785804

[16] Moosig F Czech N Mehl C Henze E Zeuner RA Kneba M. Correlation between 18-fluorodeoxyglucose accumulation in large vessels and serological markers of inflammation in polymyalgia rheumatica: a quantitative PET study. Ann Rheum Dis. (2004) 63:870–3. 10.1136/ard.2003.011692 15194587 PMC1755055

[17] Okamura K Yonemoto Y Arisaka Y Takeuchi K Kobayashi T Oriuchi N. The assessment of biologic treatment in patients with rheumatoid arthritis using FDG-PET/CT. Rheumatology. (2012) 51:1484–91. 10.1093/rheumatology/kes064 22513145

[18] Blockmans D De Ceuninck L Vanderschueren S Knockaert D Mortelmans L Bobbaers H. Repetitive 18-fluorodeoxyglucose positron emission tomography in isolated polymyalgia rheumatica: a prospective study in 35 patients. Rheumatology. (2007) 46:672–7. 10.1093/rheumatology/kel376 17114803

[19] Blockmans D de Ceuninck L Vanderschueren S Knockaert D Mortelmans L Bobbaers H. Repetitive 18F-fluorodeoxyglucose positron emission tomography in giant cell arteritis: a prospective study of 35 patients. Arthritis Rheum. (2006) 55:131–7. 10.1002/art.21699 16463425

[20] Palard-Novello X Querellou S Gouillou M Saraux A Marhadour T Garrigues F. Value of 18F-FDG PET/CT for therapeutic assessment of patients with polymyalgia rheumatica receiving tocilizumab as first-line treatment. Eur J Nucl Med Mol Imaging. (2016) 43:773–9. 10.1007/s00259-015-3287-z 26753600

[21] Cimmino MA Camellino D Paparo F Morbelli S Massollo M Cutolo M. High frequency of capsular knee involvement in polymyalgia rheumatica/giant cell arteritis patients studied by positron emission tomography. Rheumatol. (2013) 52:1865–72. 10.1093/rheumatology/ket229 23850896

[22] Rehák Z Szturz P. Comment on: FDG PET in the early diagnosis of large-vessel vasculitis. Eur J Nucl Med Mol Imaging. (2014) 41:579–80. 10.1007/s00259-013-2662-x 24435774

[23] Slart RHJA Slart RHJA Glaudemans AWJM Chareonthaitawee P Treglia G Besson FL. FDG-PET/CT(A) imaging in large vessel vasculitis and polymyalgia rheumatica: joint procedural recommendation of the EANM, SNMMI, and the PET Interest Group (PIG), endorsed by the ASNC. Eur J Nucl Med Mol Imaging. (2018) 45:1250–69. 10.1007/s00259-018-3973-8 29637252 PMC5954002

[24] Lee YH Choi SJ Ji JD Song GG Diagnostic accuracy of 18F-FDGPET or PET/CT for large vessel vasculitis: a meta-analysis. Z Rheumatol. (2016) 75:924–31. 10.1007/s00393-015-1674-2 26704559

[25] Meignan M Gallamini A Meignan M Gallamini A Haioun C. Report on the First International Workshop on interim-PET scan in lymphoma. Leuk Lymphoma. (2009) 50:1257–60. 10.1080/10428190903040048 19544140

[26] Wendling D Blagosklonov O Boulahdour H Prati C. Positron emission tomography: the ideal tool in polymyalgia rheumatica? Joint Bone Spine. (2014) 81:381–3. 10.1016/j.jbspin.2014.04.007 24962975

